# Trajectory Pathways for Depressive Symptoms and Their Associated Factors in a Chinese Primary Care Cohort by Growth Mixture Modelling

**DOI:** 10.1371/journal.pone.0147775

**Published:** 2016-02-01

**Authors:** Weng Yee Chin, Edmond P. H. Choi, Eric Y. F. Wan

**Affiliations:** 1 Department of Family Medicine and Primary Care, The University of Hong Kong, 3/F., 161 Main Street, Ap Lei Chau Clinic, Ap Lei Chau, Hong Kong; 2 School of Nursing, The University of Hong Kong, 4/F, William M. W. Mong Block 21 Sassoon Road, Pokfulam, Hong Kong; University of Geneva, SWITZERLAND

## Abstract

**Background:**

The naturalistic course for patients suffering from depressive disorders can be quite varied. Whilst some remit with little or no intervention, others may suffer a more prolonged course of symptoms. The aim of this study was to identify trajectory patterns for depressive symptoms in a Chinese primary care cohort and their associated factors.

**Methods and Results:**

A 12-month cohort study was conducted. Patients recruited from 59 primary care clinics across Hong Kong were screened for depressive symptoms using the Centre for Epidemiologic Studies Depression Scale (CES-D) and monitored over 12 months using the Patient Health Questionnaire-9 items (PHQ-9) administered at 12, 26 and 52 weeks. 721 subjects were included for growth mixture modelling analysis. Using Akaike Information Criterion, Bayesian Information Criterion, Entropy and Lo-Mendell-Rubin adjusted likelihood ratio test, a seven-class trajectory path model was identified. Over 12 months, three trajectory groups showed improvement in depressive symptoms, three remained static, whilst one deteriorated. A mild severity of depressive symptoms with gradual improvement was the most prevalent trajectory identified. Multivariate, multinomial regression analysis was used to identify factors associated with each trajectory. Risk factors associated with chronicity included: female gender; not married; not in active employment; presence of multiple chronic disease co-morbidities; poor self-rated general health; and infrequent health service use.

**Conclusions:**

Whilst many primary care patients may initially present with a similar severity of depressive symptoms, their course over 12 months can be quite heterogeneous. Although most primary care patients improve naturalistically over 12 months, many do not remit and it is important for doctors to be able to identify those who are at risk of chronicity. Regular follow-up and greater treatment attention is recommended for patients at risk of chronicity.

## Introduction

Depressive disorders are disabling, and can jeopardize social health, physical health; work performance and health-related quality of life (HRQOL)[1]. The naturalistic course for depressive disorders can be quite varied; some remit without medical intervention, whilst others experience a chronic illness course which may be resistant to treatment [2, 3]. For primary care clinicians, it is a challenge to be able to predict a patient’s illness course over time. A cohort study examining the epidemiology and outcomes of depressive disorders in primary care patients in Hong Kong found that around one in ten patients have mild to moderate depressive symptoms at the time of a primary care consultation, of whom around one in four are identified as having depression by the doctor [4]. Over 12 months, approximately 60% remit naturalistically whilst 40% continue to experience chronic depressive symptoms [5]. It was found that baseline severity of depressive symptoms and doctor detection status were not predictors for remission [5].

Given the complex nature of depressive illnesses, there have been several studies that have adopted longitudinal designs to explore symptom progression and outcomes over time. In general, these studies have identified that for many, depressive disorders are highly dynamic with fluctuating levels of symptoms which may shift between mild, moderate or severe [3]. Modelling of symptom trajectories has been used to study the longitudinal illness course from selective populations such as adolescents [6, 7], mothers who have recently given birth [8], patients with breast cancer [9] and the elderly [10], however knowledge in community-based and primary care population samples is less well established with only a few studies which have recruited subjects from primary care settings. Two studies conducted in America [10, 11] (Latino population, n = 220; the elderly n = 392) and one in Sweden [12] (elderly; n = 54) had modest and selective samples which limits the generalizability of the findings. A recent study examining the trajectory patterns for depression by Gunn et al. included 789 Australian primary care patients [13]. In this study, a five-class trajectory model was identified. Of these, two classes were dynamic, namely increasing and decreasing severity trajectories while three classes were static. Similarly, the NESDA study conducted in the Netherlands primary care setting identified five course trajectories ranging from mild severity and rapid remission to high severity and chronic course. The trajectory with the poorest outcomes trajectory differed on several characteristics from other classes including older age, earlier age of onset of depressive symptoms and lower levels of extraversion [14]. Although these study samples were larger and more representative of the primary care population, the findings might not be transferable and applicable to Chinese populations because of the differences in demographics, health beliefs, health care systems and health seeking behaviours.

Hong Kong has a pluralistic health care system, meaning that patients can seek primary health care services from a variety of settings including fee-for-service private sector as well as from government-funded general outpatient public-sector clinics. Furthermore, despite being renowned as a modern westernised centre for business and finance, its cultural roots are still firmly influenced by its Chinese origins, and many people still seek the care of Traditional Chinese Medicine (TCM) practitioners for primary health care services. The health-related behaviours of patients in Hong Kong range from those which are highly westernised, particularly in those who are younger and more educated, to patients who regularly use TCM services [15].

### Aims

To gain a better understanding of the longitudinal outcomes of primary care patients with depressive symptoms in a non-Western setting, the aims of this study were to: (1) identify the trajectory patterns for depressive symptoms in a cohort of Chinese primary care patients and; (2) explore the socio-demographic and clinical factors associated with each trajectory pattern. Greater knowledge of the factors associated with the various symptom trajectories can help inform clinicians on how to risk stratify their patients and identify who warrants more treatment attention.

## Methods

### Study design

This was a prospective cohort study to examine the natural history of depressive disorders in the Hong Kong primary care setting. The research methodology has been reported elsewhere [15–17]. In brief, it was a 12-month prospective longitudinal observational study conducted on patients recruited from primary care clinics across Hong Kong.

### Sampling and participants

To facilitate the recruitment of primary care patients, a practice-based primary care research network was established. Recruitment of doctors was via a letter of invitation to all members of the Hong Kong College of Family Physicians (N = 1500). Inclusion criteria included doctors who were primary care providers in a public-sector (government-funded) clinic or in private practice. All eligible patients who presented to the study doctor on one randomly selected day each month over one year were consecutively recruited to join the study. Field workers who provided information on the study and obtained written informed consent approached patients in the doctor’s waiting room. Patients were excluded if they were (1) aged < 18 years, (2) could not understand English, Cantonese or Mandarin, (3) had cognitive or communication difficulties, (4) had already been recruited to the study or (5) did not consult the study doctor. Subjects completed a questionnaire containing items on socio-demographics, the Patient Health Questionnaire -9 (PHQ-9), the Centre for Epidemiologic Studies Depression Scale (CES-D), the Short Form-12 Health Survey version 2 (SF-12 v2), and recent health service use prior to their index doctor consultation visit. At the completion of the baseline questionnaire, all subjects were invited to participate in the 12-month longitudinal study. Those who agreed by providing their names and contact numbers were subsequently followed up by telephone interview at 3, 6 and 12 months. The study design and flowchart is shown in **S1 Fig.**

### Instruments

The CES-D is a 20-item self-report scale which measures the level of depressive symptoms experienced in the past week [18]. It has been validated in community and primary care populations in Hong Kong, and has good test-retest reliability [19–21]. Total sum scores can range from 0 to 60 with scores of 16 to 26 considered indicative of mild depression and scores of >27 indicative of more severe depression. In this study, a CES-D score ≥16 was used to identify patients with depressive symptoms at baseline interview and for inclusion in the data analysis.

The PHQ-9 is a 10-item self-report depression scale [22] which scores each of the nine Diagnostic and Statistical Manual of Mental Disorders, 4th Edition (DSM-IV) criteria for a diagnosis of major depression episode plus an extra item on functional difficulties as a result of the depressive symptoms. It has been validated for screening use in the Hong Kong primary care setting [23] as well as for telephone administration [24]. The PHQ-9 score can be categorized by severity of symptoms (score 1–4 minimal, 5–9 mild, 10–14 moderate, 15–19 moderately severe, 20–27 severe) [25]. The PHQ-9 was administered at baseline, 3, 6 and 12-month and the sum scores used as an outcome measure. Only subjects who had a valid PHQ-9 score available at two or more time points were included for data analysis.

The SF-12 v2 is a 12-item abbreviated version of the SF-36 Health Survey that assesses functional health and wellbeing from a patient’s perspective and was administered at baseline. The SF-12v2 generates two norm-based summary scores: a mental component score (MCS) and a physical component score (PCS), with higher scores indicting better health status. In a general population the mean score on each component is 50. The SF-12 v2 has been validated and normed in the Hong Kong general population [26, 27]. In this study, the SF-12 v2 was used to measure (1) general health, and (2) functioning affected by pain, (3) overall physical health-related quality of life and (4) overall mental health-related quality of life.

For questions on past health, all subjects were asked if he/she had ever been diagnosed by a registered western doctor to have depression or other mental illness, and if he/she had ever been diagnosed by a doctor to have hypertension, diabetes mellitus, heart disease, stroke, chronic respiratory disease (such as asthma, chronic bronchitis and emphysema), arthritis or other chronic rheumatism, chronic pain and other major diseases.

For questions on recent health service utilization subjects were asked: (1) if he/she had seen a western doctor in the past 4 weeks for any medical problem, or (2) if he/she had seen a traditional Chinese medicine practitioner in the past 4 weeks for any medical problem

### Data analysis

Following the analytical methods used by Gunn et al [13], growth mixture modelling (GMM) was used to identify the latent trajectory classes based on PHQ-9 scores obtained at four time points (baseline, 3 months, 6 months and 12 months) [13, 28, 29]. Standard quadratic trajectories were developed to investigate the change in PHQ-9 scores over time. Variances of slope and quadratic factors were fixed at zero for the analysis. 5000 random sets of start values were requested for each model and the 100 best retained for final optimization. All models converged on a replicated solution and could confidently be assumed to obtain the best maximum likelihood. The selection strategy for number of classes is still controversial [30–32]. Goodness-of-fit statistics including Akaike Information Criterion (AIC) [33], Consistent AIC [34], Bayesian Information Criterion (BIC) [35] and Sample Adjusted BIC [36] were used with smaller values indicating a better fit of the model. The standard guidance suggests that there is a strong evidence to reject k-1-class model by k-class model if the BIC difference between models is larger than 10 [37]. The Elbow plot of the indicators of Goodness-of-fit statistics was generated and indicated diminishing improvements from estimating additional classes if the indicators tended to decrease in a less pronounced fashion. The classification accuracy of the model was summarized by using Entropy [38, 39], ranging from 0 to 1. A higher value indicated less classification error, and the model had adequate classification quality if the value was ≥0.8 [40]. The Lo-Mendell-Rubin adjusted likelihood ratio test [41] was used to compare a k-class model with a k-1-class model. A significant p-value illustrated that the k-class model had significant improvement over the k-1-class model.

The command ‘r3step’ in MPlus [42, 43], which is the 3 steps method developed in Vermunt 2010 [44], was used to investigate the predictors of group membership. In general, the class model is estimated in a first step using only latent class indicator variables. In the second step the most likely class variable is created using the latent class posterior distribution obtained during the first step. In the third step the most likely class is regressed on predictor variables by multinomial logistic regression taking into account the misclassification in the second step. Factors included in the regression analysis were: gender, age, marital status, working status, number of co-morbidities, recent Western Medicine service use; recent Traditional Chinese Medicine service sue, self-reported past history of depression, self-rated health status, and functioning affected by pain. Multiple imputation was used to replace missing values. Each missing value was imputed ten times, and for each of the ten imputed datasets, the same analysis was performed with the five sets of results combined using Rubin’s combination rules [45].

All statistical analyses were performed using Mplus Version 7 software program [29] and SPSS version 21.0 (SPSS, IBM Inc., Chicago, Illinois, USA). All significance tests were two-tailed and findings with a p-value less than 0.05 were considered statistically significant.

### Ethics approval

This study was approved by the Institutional Review Board of the University of Hong Kong/Hospital Authority Hong Kong West Cluster, the Research Committee of Hong Kong Sanatorium and Hospital, the Research Ethics Committee for Hong Kong Hospital Authority Kowloon East and Kowloon Central, and the Joint Chinese University of Hong Kong and Hong Kong Hospital Authority New Territories East Clinical Ethics Review Committee.

## Results

59 primary care physicians (PCPs) participated: 81% worked in private settings; 19% worked in public sector Government Out-patient Clinics (GOPC) in proportionate alignment with the delivery of primary care in Hong Kong. A total of 10,179 subjects completed the baseline questionnaire (response rate of 81.0%) of which 4,358 subjects agreed to participate in the longitudinal follow-up (response rate of 42.8%). Response rates at each follow-up time point was 70.20% (12 weeks); 77.14% (26 weeks); and 80.4% (52 weeks). Amongst the 4,358 subjects who consented to longitudinal follow up, 3,920 (89.9%) completed the CES-D and PHQ-9 scales at baseline. Of these, 721 (18.4%) subjects scored ≥ 16 on the CES-D at baseline and completed at least one PHQ-9 follow-up interview in the subsequent 3 to 12 months and were included in the GMM analysis (as shown in **S1 Fig**).

A comparison of those who consented to follow-up and those who refused are shown in **Table 1**. Compared to those who refused follow up, the mean age of the cohort participants were slightly older (49.6 years vs 48.6 years); slightly higher proportion were married (63.6%vs 56.5%); slightly more had only attained primary school level education (18.4% vs. 14.2%); slightly higher proportion had a positive PHQ-9 screening score >9 (11.8% vs 9.7%). The baseline characteristics for subjects who were included in the screening cohort sample (N = 3546), the selected cohort sample (N = 721) and the non-selected cohort sample (N = 2825) are shown in **Table 2**. All characteristics except for number of co-morbidities were significantly different between the selected cohort (n = 721) and non-selected cohort (n = 3199). Subjects in the selected cohort were more likely to be female, at a younger age, not married, to have a lower household monthly income, to have a higher educational attainment, to be in active employment. The mean scores of CES-D and PHQ-9 in the selected cohort were three times higher than the non-selected cohort. The SF-12v2 PCS and MCS in the selected cohort were also significantly lower (indicating a poorer health-related quality of life) than those in the non-selected cohort. More subjects in the selected cohort reported poorer general health and functioning affected by pain.

**Table 1:** Comparisons between cohort study participants and non-participants.

Subject characteristics	Refused Follow-up (n = 5,821)	Consented to Follow-up (n = 4,358)	Total (N = 10,179)	P-value
Gender (n, %)				0.713
Male	2,339 (40.2%)	1,803 (41.4%)	4,142 (40.7%)	
Female	3,233 (55.5%)	2,530 (58.1%)	5,763 (56.6%)	
Not indicated	249 (4.3%)	25 (0.6%)	274 (2.7%)	
Age (yr)	48.59 ± 18.74 (range 18–103)	49.59 ± 17.11 (range 18–94)	49.03 ± 18.05 (range 18–103)	0.006*
18–24 years	420 (7.2%)	269 (6.2%)	689 (6.8%)	
25–24 years	1,127 (19.4%)	747 (17.1%)	1,874 (18.4%)	
35–44 years	978 (16.8%)	741 (17.0%)	1,719 (16.9%)	
45–54 years	960 (16.5%)	817 (18.7%)	1,777 (17.5%)	
55–64 years	768 (13.2%)	834 (19.1%)	1,602 (15.7%)	
65+ years	1,183 (20.3%)	888 (20.4%)	2,071 (20.3%)	
Not indicated	385 (6.6%)	62 (1.4%)	447 (4.4%)	
Household monthly income (n, %)				0.751
≤HK$5000	683 (11.7%)	560 (12.8%)	1,243 (12.2%)	
HK$5,001-HK$10,000	405 (7.0%)	356 (8.2%)	761 (7.5%)	
HK$10,001-HK$20,000	989 (17.0%)	826 (19.0%)	1,815 (17.8%)	
HK$20,001-HK$30,000	828 (14.2%)	705 (16.2%)	1,533 (15.1%)	
HK$30,001-HK$40,000	557 (9.6%)	516 (11.8%)	1,073 (10.5%)	
>HK$40,000	1,165 (20.0%)	994 (22.8%)	2,159 (21.2%)	
Not indicated	1,194 (20.5%)	401 (9.2%)	1,595 (15.7%)	
Marital status (n, %)				<0.001*
Single	1,618 (27.8%)	1,081 (24.8%)	2,699 (26.5%)	
Married	3,288 (56.5%)	2,771 (63.6%)	6,059 (59.5%)	
Widowed	443 (7.6%)	330 (7.6%)	773 (7.6%)	
Separated/ divorced	163 (2.8%)	163 (3.7%)	326 (3.2%)	
Not indicated	309 (5.3%)	13 (0.3%)	322 (3.2%)	
Education level (n, %)				<0.001*
No formal schooling	501 (8.6%)	274 (6.3%)	775 (7.6%)	
Primary	829 (14.2%)	800 (18.4%)	1,629 (16.0%)	
Secondary	2,263 (38.9%)	1,864 (42.8%)	4,127 (40.5%)	
Tertiary	1,920 (33.0%)	1,405 (32.2%)	3,325 (32.7%)	
Not indicated	308 (5.3%)	15 (0.3%)	323 (3.2%)	
District of residence (n, %)				<0.001*
Hong Kong Island	2,212 (38.0%)	1,873 (43.0%)	4,085 (40.1%)	
Kowloon	1,209 (20.8%)	1,034 (23.7%)	2,243 (22.0%)	
New Territories	2,042 (35.1%)	1,431 (32.8%)	3,473 (34.1%)	
Not indicated	358 (6.2%)	20 (0.5%)	378 (3.7%)	
PHQ-9 screening				0.001*
PHQ+ve (score >9)	563 (9.7%)	516 (11.8%)	1079 (10.6%)	
PHQ-ve (score ≤9)	5,048 (86.7%)	3,743 (85.9%)	8791 (86.4%)	
Not categorized due to incompletion	210 (3.6%)	99 (2.3%)	309 (3.0%)	

* Significant differences (P<0.05) between groups by independent t-test or by Chi-square test, as appropriate

**Table 2:** Baseline characteristics of the selected cohort.

	Screening sample (N = 3546)	Selected Cohort (N = 721)	Excluded Subjects (N = 2825)	P-value
Gender (n, %)				0.038*
Male	1,467 (41.5%)	274 (38.2%)	1,193 (42.4%)	
Female	2,064 (58.5%)	443 (61.8%)	1,621 (57.6%)	
Age (Mean ± SD)	49.1 ± 17.1	44.0 ± 16.2	50.4 ± 17.0	<0.001*
PHQ-9 CES-D	4.4 ± 4.4	9.8 ± 5.0	3.0 ± 2.8	<0.001*
SF12v2—PCS	9.6 ± 9.5	24.9 ± 8.3	5.8 ± 4.6	<0.001*
SF12v2—MCS	47.7 ± 9.0	44.7 ± 10.8	48.5 ± 8.3	<0.001*
Marital status (n, %)	53.0 ± 11.1	39.6 ± 11.5	56.4 ± 8.0	<0.001*
Married	2,248 (63.6%)	374 (52.2%)	1,874 (66.4%)	<0.001*
Others	1,289 (36.4%)	342 (47.8%)	947 (33.6%)	
Working Status (n, %)				0.012*
Employed	1,738 (62.2%)	474 (66.8%)	2,212 (63.1%)	
Others	1,055 (37.8%)	236 (33.2%)	1,291 (36.9%)	
Number of co-morbidity (n, %)				0.396
None	1,599 (45.6%)	324 (45.8%)	1,275 (45.5%)	
One	953 (27.2%)	176 (24.9%)	777 (27.8%)	
Two or more	955 (27.2%)	207 (29.3%)	748 (26.7%)	
Seen a western doctor in past 4 weeks (n, %)				<0.001*
No	1,710 (48.9%)	256 (36.2%)	1,454 (52.2%)	
Once or twice	1,376 (39.4%)	314 (44.4%)	1,062 (38.1%)	
More than twice	409 (11.7%)	138 (19.5%)	271 (9.7%)	
Seen a TCM practitioner in past 4 weeks (n, %)				<0.001*
No	2,902 (83.7%)	555 (78.9%)	2,347 (84.9%)	
Once or more	567 (16.3%)	148 (21.1%)	419 (15.1%)	
Diagnosis of depression by doctor at baseline (n, %)				<0.001*
No	3,138 (92.5%)	559 (79.5%)	2,579 (95.9%)	
Yes	254 (7.5%)	144 (20.5%)	110 (4.1%)	
Self-rated health status				<0.001*
Good to excellent	1,298 (46.1%)	156 (21.7%)	1,454 (41.1%)	
Poor to fair	1,516 (53.9%)	564 (78.3%)	2,080 (58.9%)	
Functioning affected by pain the past month				<0.001*
Not at all to a little bit	2,079 (73.8%)	302 (42.1%)	2,381 (67.4%)	
Moderately to extremely	738 (26.2%)	416 (57.9%)	1,154 (32.6%)	

† Denominators vary owing to missing data

‡ Professional psychological treatment or counseling including doctor, psychologists, social workers or other

* Significant differences (P<0.05) between groups by independent t-test or by Chi-square test, as appropriate

All study measures showed good internal consistency. The Cronbach’s alpha values of the CES-D, the PHQ-9 and the SF12v2 for the present sample were 0.73, 0.77 and 0.83, respectively.

The possible trajectory models for depressive symptoms with parameter estimates of a quadratic model and Goodness-of-fit statistics are shown in **Table 3**. Eight models were initially identified however the seven-class model as the best fitting model because it had the lowest BIC (16254.44), the lowest consistent AIC (16286.44), second lowest AIC (16107.86) and sample adjusted BIC (16152.84). The value of entropy of the seven-class model >0.8 indicated that the seven-class model had good quality of classification. The results of Lo-Mendell-Rubin adjusted likelihood ratio tests indicated a statistically significant difference between the six-class and seven-class models (p-value = 0.043), suggesting that the seven class mode had a better fit, whilst the result of Lo-Mendell-Rubin adjusted likelihood ratio tests indicated a statistically insignificant difference between the seven-class and eight-class models (p-value = 0.144), suggesting that eight-class model failed to improve the fit. **S2 Fig** shows the Elbow plot of Goodness-of-fit statistics. The indicators of Goodness-of-fit statistics tended to level off after the seven-class model, suggesting that the best fitting model was the seven-class model. Hence, seven classes were retained as the best fitting model.

**Table 3:** Goodness of fit statistics and parameter estimates for quadratic trajectories of depressive.

Groups	Subjects	I	SE	P-value	S	SE	P-value	Q	SE	P-value	AIC	Consistent AIC	BIC	Sample Adjusted BIC	Entropy
1	721	9.299	0.176	<0.001*	-1.236	0.053	<0.001*	0.07	0.004	<0.001*	16449.3	16493.94	16485.94	16460.541	
2	614	8.921	0.248	<0.001*	-1.357	0.06	<0.001*	0.072	0.005	<0.001*	16323.5	16390.62	16378.62	16340.516	0.819
	107	11.455	0.867	<0.001*	-0.622	0.227	0.006*	0.056	0.02	0.005*					
3	520	7.875	0.429	<0.001*	-1.267	0.136	<0.001*	0.071	0.01	<0.001*	16265.58	16354.87	16338.87	16288.07	0.756
	80	15.339	1.762	<0.001*	-2.114	1.182	0.074	0.092	0.083	0.267					
	121	11.363	0.68	<0.001*	-0.646	0.195	0.001*	0.058	0.016	<0.001*					
4	492	7.737	0.464	<0.001*	-1.351	0.12	<0.001*	0.076	0.008	<0.001*	16203.57	16315.18	16295.18	16231.676	8.07
	14	17.18	1.966	<0.001*	0.369	0.954	0.699	-0.04	0.077	0.602					
	88	14.28	1.708	<0.001*	-1.469	1.045	0.16	0.051	0.072	0.479					
	128	10.578	0.719	<0.001*	-0.817	0.206	<0.001*	0.07	0.018	<0.001*					
5	112	10.279	1.222	<0.001*	-0.858	0.252	0.001*	0.079	0.031	0.012*	16169.5	16303.43	16279.43	16203.224	0.813
	477	7.497	0.248	<0.001*	-1.263	0.079	<0.001*	0.071	0.006	<0.001*					
	76	13.096	1.591	<0.001*	-0.607	0.401	0.13	0.002	0.037	0.961					
	45	17.413	1.393	<0.001*	-3.646	0.704	<0.001*	0.202	0.051	<0.001*					
	11	17.489	1.958	<0.001*	0.472	0.871	0.588	0.045	0.066	0.491					
6	147	9.144	0.981	<0.001*	-1.232	0.197	<0.001*	0.088	0.015	<0.001*	16135.53	16291.79	16263.79	16174.879	0.81
	56	10.513	1.307	<0.001*	-0.882	0.409	0.031*	0.093	0.041	0.023*					
	11	18.008	1.543	<0.001*	-0.101	1.114	0.928	0.01	0.094	0.917					
	53	13.898	1.383	<0.001*	-0.72	0.621	0.246	0.025	0.039	0.518					
	384	7.283	0.361	<0.001*	-1.317	0.115	<0.001*	0.071	0.008	<0.001*					
	71	14.424	1.632	<0.001*	-1.811	0.981	0.065	0.066	0.07	0.346					
7	375	7.274	0.316	<0.001*	-1.293	0.117	<0.001*	0.068	0.008	<0.001*	16107.86	16286.44	16254.44	16152.835	0.817
	136	8.612	0.931	<0.001*	-1.236	0.214	<0.001*	0.091	0.016	<0.001*					
	19	9.459	2.373	<0.001*	-0.992	0.662	0.134	0.127	0.046	0.006*					
	66	11.942	1.779	<0.001*	-0.706	0.433	0.103	0.057	0.044	0.2					
	53	13.878	1.503	<0.001*	-1.016	0.617	0.1	0.034	0.049	0.488					
	64	15.038	1.879	<0.001*	-2.228	1.089	0.041*	0.093	0.08	0.244					
	8	18.437	1.358	<0.001*	0.408	0.879	0.642	-0.037	0.069	0.594					
8	8	18.49	1.435	<0.001*	0.489	1.098	0.656	-0.043	0.089	0.629	16090.94	16291.85	16255.85	16141.536	0.816
	128	8.346	0.784	<0.001*	-1.201	0.253	<0.001*	0.09	0.02	<0.001*					
	55	13.866	2.0	<0.001*	-1.08	0.566	0.056	0.044	0.038	0.253					
	38	12.461	1.21	<0.001*	-0.468	0.541	0.387	-0.035	0.039	0.363					
	22	9.411	2.528	<0.001*	-1.013	0.647	0.117	0.127	0.047	0.006*					
	36	17.579	1.423	<0.001*	-3.799	0.557	<0.001*	0.209	0.041	<0.001*					
	374	7.232	0.245	<0.001*	-1.271	0.082	<0.001*	0.067	0.006	<0.001*					
	62	12.201	1.645	<0.001*	-0.668	0.373	0.073	0.052	0.036	0.149					

Note: I = Intercept; S = Linear slope; Q = Quadratic slope; SE = Standard error; AIC = Akaike Information Criterion; BIC = Bayesian Information Criterion

* Significant if p<0.05

The symptom trajectories for the seven-class model are shown in **S3 Fig**. Based on the baseline PHQ-9 scores, three classes had mild depressive symptoms (mean PHQ-9 score 5–9) at baseline, two classes had moderate depressive symptoms (mean PHQ-9 score 10–14) at baseline and two classes had moderately severe depressive symptoms (mean PHQ-9 score 15–19) at baseline. Just over half of the cohort were categorised into **Class 1** (n = 375, 52.0%) with a trajectory that started with mild depressive symptoms at baseline followed by a naturalistically recovery to minimal symptoms (PHQ-9 <5) over 12 months. **Class 2** (n = 136, 18.9%) was characterized by a U-shaped trajectory, with mild depressive symptoms at baseline that dropped to minimal at 6 months but reverted to mild severity at 12 months. **Class 3** (n = 19, 2.6%) had a deteriorating symptom trajectory, increasing from the upper limits of mild symptoms at baseline to moderately severe symptoms at 12-months. **Class 4** (n = 66, 9.2%) had a U-shaped trajectory, similar to that of Class 2, however at a higher severity of symptoms with baseline and 12-month symptoms at approximately the same levels. **Class 5** (n = 53, 7.4%) had a gradually decreasing trajectory featuring moderate level of symptoms at baseline dropping to mild severity of symptoms at 6-months, and a continuing reduction in symptoms up to 12-months. **Class 6** (n = 64, 8.9%) had a downward trajectory that started with moderately severe depressive symptoms at baseline followed by a recovery to minimal symptoms over 12 months. **Class 7** (n = 8, 1.1%) had a stable but a high symptom trajectory, featuring depressive symptoms that remained at moderately severe levels for the entire 12-months.

Comparisons of the baseline characteristics of the subjects in the seven trajectory classes by multinomial logistic regressions are shown in **Table 4**.

**Table 4:** Comparisons of the demographic and clinical characteristics of the seven trajectory paths by multinomial logistic regressions.

	Class 2 (n = 136)	Class 3 (n = 19)	Class 4 (n = 66)	Class 5 (n = 53)	Class 6 (n = 64)	Class 7(n = 8)	Group difference
	Estimate (SE)	Estimate (SE)	Estimate (SE)	Estimate (SE)	Estimate (SE)	Estimate (SE)	
Gender (Male)							
Female	-0.88 (0.39)*	0.32 (0.35)	0.31 (0.35)	0.34 (0.27)	0.37 (0.23)	7.09 (2.24)*	7>1,2,3,4,5,6; 1,6>2
Age (Mean ± SD)	14.61 (5.39)*	1.20 (0.44)*	1.20 (0.44)*	1.20 (0.44)*	1.20 (0.44)*	1.27 (0.47)*	2>7>3,4,5,6>1
Marital status (Married)							
All others (Single/Separated/Divorced/Widowed)	0.02 (0.20)	0.13 (0.50)	0.05 (0.28)	0.07 (0.30)	0.06 (0.25)	-1.20 (0.58)*	1,3,4,5,6>7
Working Status (Employed)							
Other (Unemployed, retired, housemaker or student)	0.10 (0.12)	0.11 (0.40)	0.07 (0.28)	0.10 (0.40)	0.06 (0.27)	-0.77 (0.43)	2,3,4,5,6>7
Co-morbidity (No)							
Yes	1.89 (0.43)*	1.79 (0.63)*	1.86 (0.39)*	1.79 (0.55)*	1.80 (0.51)*	1.91 (0.40)*	2,3,4,5,6,7>1
Seen a western doctor in past 4 weeks (No)							
Yes	7.47 (2.84)*	-0.84 (0.33)*	-0.75 (0.15)*	-0.67 (0.35)	-0.68 (0.33)*	0.49 (0.29)	2>7>1,3,4,5,6;1>3,4,6
Seen a TCM practitioner in past 4 weeks (No)							
Yes	13.53 (5.10)*	0.03 (0.23)	0.04 (0.21)	0.05 (0.26)	0.00 (0.18)	-2.43 (8.29)	2>1,3,4,5,6,7
Self-reported doctor diagnosed depression or other mental illness (No)							
Yes	-1.84 (0.95)	-2.47 (8.18)	0.08 (0.30)	0.19 (0.64)	0.15 (0.50)	10.56 (3.22)*	7>1,2,3,4,5,6;4,5,6>2
Self-rated health status (Good to excellent)							
Poor to fair	-2.02 (1.01)*	1.17 (9.07)	-1.18 (1.69)	-1.32 (0.84)	-1.39 (0.60)*	5.79 (2.53)*	7>1,2,5,6;1,5>2;1>6
Functioning affected by pain the past month (Not at all to a little bit)							
Moderately to extremely	0.05 (0.12)	-0.03 (0.23)	0.02 (0.26)	-0.04 (0.17)	0.01 (0.25)	-0.90 (1.02)	

* Significant differences (P<0.05) comparing with Class 1 (n = 375) by multinomial logistic regression

Subjects in Class 1 were less likely to have co-morbidity and more likely to be younger than subjects in all other classes. There were more likely to have seen a western doctor in past 4 weeks than Class 3, 4, and 6. They were less likely to rate their health as poor to fair than subjects in Class 2 and 6.

Subjects in Class 2 were more likely to be older aged compared with subjects in other groups. They were less likely to be female than subjects in Class 1 and 6. They were more likely to have seen a western doctor or a TCM practitioner in past 4 weeks than subjects in all other groups. They were less likely to have self-reported doctor diagnosed depression or other mental illness than subjects in Class 4, 5, and 6.

Subjects in Class 7 were more likely to be female, less likely to be currently married, less likely to be in paid employment, more likely to have seen a western doctor in past 4 weeks, more likely to have self-reported doctor diagnosed depression or other mental illness and more likely to rate their health as “poor to fair”.

## Discussion

This was the first study to explore 12-month trajectories for depressive symptoms in a Chinese primary care patient population. Using growth mixture modelling, seven distinct depression trajectories were identified. This differed from the findings of earlier studies conducted in Australian and Dutch primary care patients which both identified five-class models [13, 14]. Compared with the trajectory patterns identified in the Australian and Dutch studies, the trajectories in the Hong Kong setting appeared to be more heterogeneous and dynamic. The pattern variation is potentially due to the differences in demography, health service delivery and health or cultural beliefs between Chinese and Western populations.

Whilst Class 1, 2 and 3 all had baseline depressive scores in the ‘mild’ severity range, three different 12-month trajectory patterns were observed with Class 1 improving, Class 2 remaining stable and Class 3 deteriorating. Similar to studies in Australia [13, 19] and in the Netherlands [14], the present study found that the most common pattern (Class 1) representing just over half of the cohort, was of mild depressive symptoms which quickly resolved within 3 months, and remained stable with minimal depressive symptoms up to 12 months. The Class 1 trajectory course likely reflects those with sub-clinical depression and an understanding of the factors associated with this group may be relevant to population health [13, 46]. Subjects with this trajectory pattern were generally younger, with better general health and were less likely to have chronic disease co-morbidity. This result was consistent with previous studies that have found an association between general health status and depression [47, 48]. There were differences in self-reported health service use between Class 1, 2 and 3 with more patients in Class 1 and 2 having reported to receive western medicine or Chinese medicine services. It is possible that their interventions helped to prevent deterioration of symptoms that was observed in Class 3 subjects. It is possible that those in Class 3 were less health conscious or had more barriers to seeking medical care. There were also differences in age. Subjects in Class 2 were more likely to be older than those in Class 3. Previous studies have suggested that age may have a protective effect on mental health in different patient populations [4, 49]. First, depressive symptoms decline with the increase of age because of a substantial reduction in exposure to risk factors. The most important one is work-related stressors [26, 50]. Secondly, ageing of the brain may affect emotional responsiveness [50]. Older individuals are less likely to attend to and remember negative emotional material [51]. Third, as people age, most become more adaptive. Older patients develop better coping skills on chronic conditions.

Class 4 and 5 both had baseline depressive symptoms scores in the ‘moderate’ severity range, however Class 5 showed improvement whilst Class 4 remained stable over 12 months. Multinomial analysis found no difference in risk factors between these groups. This may have been because we only included ten factors in the multinomial regression and may not have included factors that can differentiate between the two classes. Alternately, Class 4 and Class 5 may potentially belong to the same trajectory group with significant within-group heterogeneity.

Class 6 and 7 both had baseline depressive symptoms scores in the ‘moderately-severe’ severity range. Whilst Class 6 had a good prognostic trajectory with remission over 12 months, those in Class 7 had very poor outcomes. Subjects in Class 7 were more likely to have a past history of depression or other mental illness, more likely to have poorer self-rated health status and more likely to have seen a western doctor in past 4 weeks, suggesting that Class 7 subjects had a far more severe spectrum of illness, which may have been resistant to treatment. It is possible that these depressive symptoms were part of a psychiatric diagnosis associated with poorer prognosis such as a mixed anxiety and depressive disorder, personality disorder, bipolar disorder or a psychotic disorder. Of note, this group formed only 1% of the cohort and were likely to represent the small group of patients encountered in primary care that are disabled by very high levels of mental morbidity. In Class 6, despite reporting moderately severe levels of depressive symptoms at baseline, symptom resolution occurred reasonably rapidly with remission observed over 12 months. It is possible that as a result of having such high levels of symptoms, these subjects were able to receive prompt treatment. Alternately, the high baseline symptoms may have been due to an acute reaction to a life event such as a bereavement or job loss, and symptoms subsided as people learnt to re-adjust.

### Clinical implications

In terms of clinical application, given that a depressive illness course can be heterogeneous, the trajectory model provides information on how to stratify patients who may be at risk for non-remission over 12 months. It also provides information on how to further risk stratify the three sub-types of patients who experience chronicity: those who improve but fail to remit, those who fail to improve, and those who actually deteriorate. As symptoms can progress differently for varying individuals, our findings suggest that subjects who are female, who are not married, who are not in active employment, which have multiple co-morbidities, who have poorer self-rated general health warrant more treatment attention because they are at higher risk of chronicity.

Furthermore, whilst all three sub-types fail to remit over 12-months, the group whose symptoms are worse at 12 months than at baseline (Class 3) seem to be at greatest risk. These patients appear to be younger and use health services infrequently. They potentially represent a group who have very poor help-seeking behaviours and regular follow-up is recommended to monitor their progress and introduce ways to optimise their mental health.

### Strengths and limitations

There were a number of strengths of the present study. First, it was a naturalistic study, which tracks the natural course of symptoms and better captures the ‘real world’ situation. As subjects were not participants of clinical trials that can interfere or distort the symptom trajectories, the findings can be more easily applied in real clinical settings. Second, the subjects had a wide spectrum of depressive symptom severity and wide range of socio-demographic characteristics. Moreover, subjects were recruited from primary care settings and the findings should be more transferable and generalizable to the general population than patients recruited from psychiatric clinics of hospital in-patient settings. Third, the primary outcome was measured by the PHQ-9, which is commonly used to depressive symptoms in primary care.

There were also several limitations. The depressive symptoms were measured using a symptom scoring self-report instrument and may be affected by recall bias. The sample sizes in some classes were very small, and may not have been sufficiently powered to detect the association between factors and trajectories. Data on treatment and treatment adherence such as number of follow-up visits were not collected and could not be reported.

## Conclusions

The naturalistic course for depressive illnesses cannot easily be predicted based on the measurement of symptoms at a single time point. Whilst patients with mild symptoms appear to have fewer co-morbidities and a more predictable illness course, patients with moderate to severe symptoms are more heterogeneous and their outcomes may be quite varied. In clinical practice, a more pro-active approach to treatment is recommended for patients with moderate to severe symptoms of depression, particularly those who have poorer general health. Scheduling of regular follow-up is recommended for younger patients with moderate depressive symptoms, especially those who use health services infrequently.

Growth mixture modelling provides an informative method for examining and summarizing longitudinal symptom paths for depressive symptoms in large population settings; information regarding demography, self-reported health status, health utilization pattern, and co-morbidity may be used for risk stratification and to predict longitudinal prognosis. Further cross-cultural studies are needed to understand and compare the trajectories of depressive symptoms across different cultural settings. As only ten factors were included in the multinomial logistic regression analysis, future studies with larger sample sizes are needed to explore the interaction of more variables such as psychological attributes of study subjects and other modifiable environmental risk factors. This study followed subjects for 12-months. Studies with longer follow-up duration are needed to examine more longitudinal outcomes such as time to relapse and factors associated with relapse.

## Supporting Information

S1 Fig Study design and sampling method. 10,179 Subjects were consecutively recruited from the waiting rooms of 59 primary care clinicians one day per month over a 12 month data collection period and asked to complete a baseline questionnaire containing the CES-D, the PHQ-9 and the SF-12v2, as well as items on socio-demography and co-morbidity. Study doctors provided information on whether they thought the patient had a depressive disorder. Subjects who completed the baseline study were invited to participate in a 12-month follow-up study. Those who consented (N = 4,358) were monitored by telephone interview at 12, 26 and 52 weeks. Inclusion criteria for trajectory modelling included: PHQ-9 and CES-D scores available at baseline; PHQ-9 score available at 12, 26 or 52 weeks; baseline CES-D score ≥ 16 (N = 721).
(PDF)

S2 Fig Figure of the Elbow plot of Goodness-of-fit statistics. The seven-class model has the lowest BIC (13597.25) and Consistent AIC (13629.25), and the second lowest AIC (13450.67) and Sample Adjusted BIC (13495.64). The indicators of Goodness-of-fit statistics tended to level-off after seven-class model, suggesting that the best fitting model was the seven-class model. Hence, seven classes were retained as the most better-fit model.
(PDF)

S3 Fig Figure of the trajectories of the seven-class model over 1 year. Based on the baseline PHQ-9 scores, one class started with mild depressive symptoms, four classes started with moderate depressive symptoms and two classes started with moderately severe depressive symptoms.
(PDF)
