# Supplementary figures and images for: Trajectory Pathways for Depressive Symptoms and Their Associated Factors in a Chinese Primary Care Cohort by Growth Mixture Modelling
Source: PLoS One. 2016 Feb 1;11(2):e0147775. doi: 10.1371/journal.pone.0147775 (PMC4734622; doi:10.1371/journal.pone.0147775)

Figure 1: Study design and sampling method

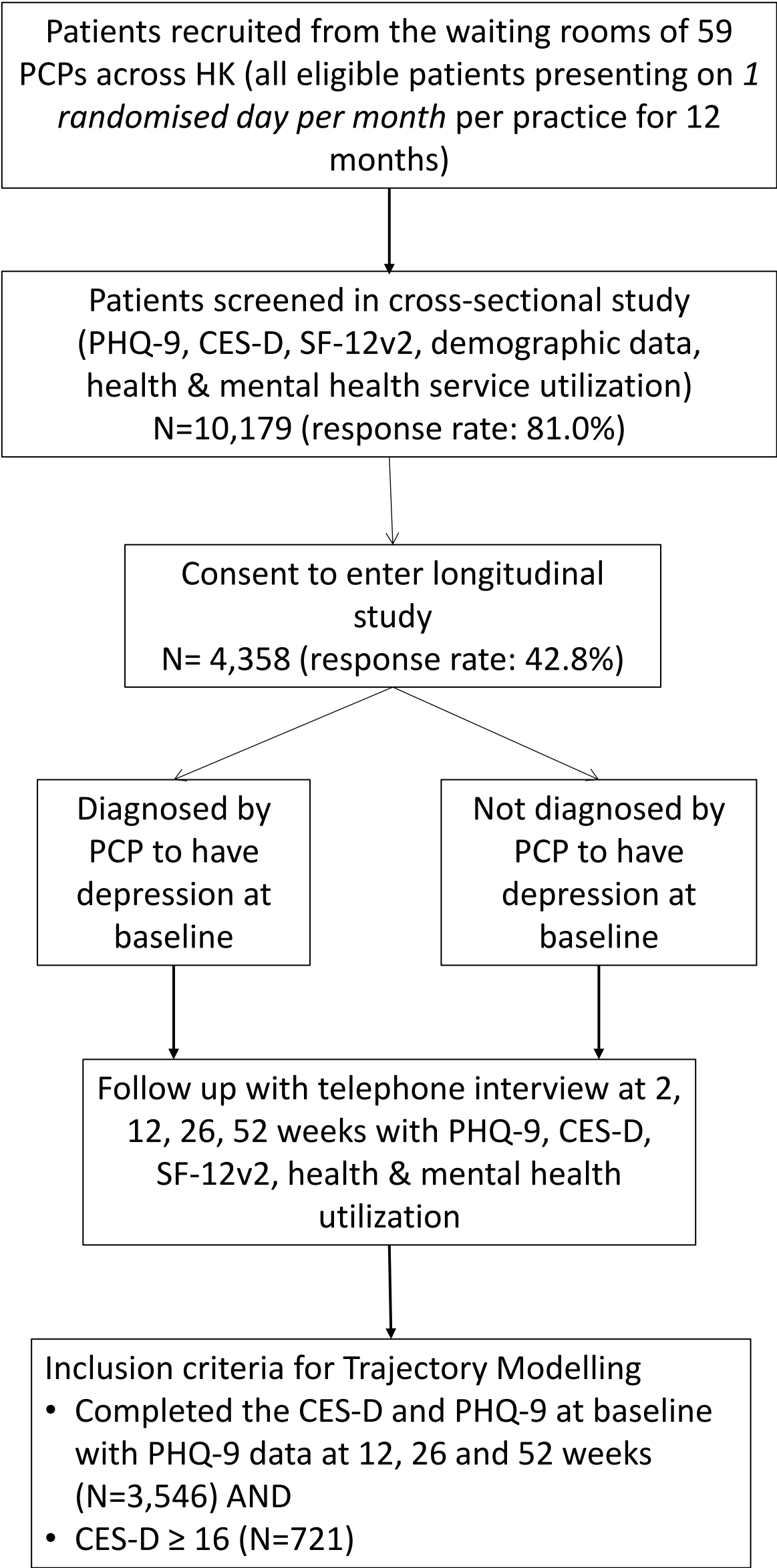

Supplement: S1 Fig — 10,179 Subjects were consecutively recruited from the waiting rooms of 59 primary care clinicians one day per month over a 12 month data collection period and asked to complete a baseline questionnaire containing the CES-D, the PHQ-9 and the SF-12v2, as well as items on socio-demography and co-morbidity. Study doctors provided information on whether they thought the patient had a depressive disorder. Subjects who completed the baseline study were invited to participate in a 12-month follow-up study. Those who consented (N = 4,358) were monitored by telephone interview at 12, 26 and 52 weeks. Inclusion criteria for trajectory modelling included: PHQ-9 and CES-D scores available at baseline; PHQ-9 score available at 12, 26 or 52 weeks; baseline CES-D score ≥ 16 (N = 721). (PDF) [file pone.0147775.s001.pdf]

**Figure 3: Figure of the trajectories of the seven-class model over 1 year**

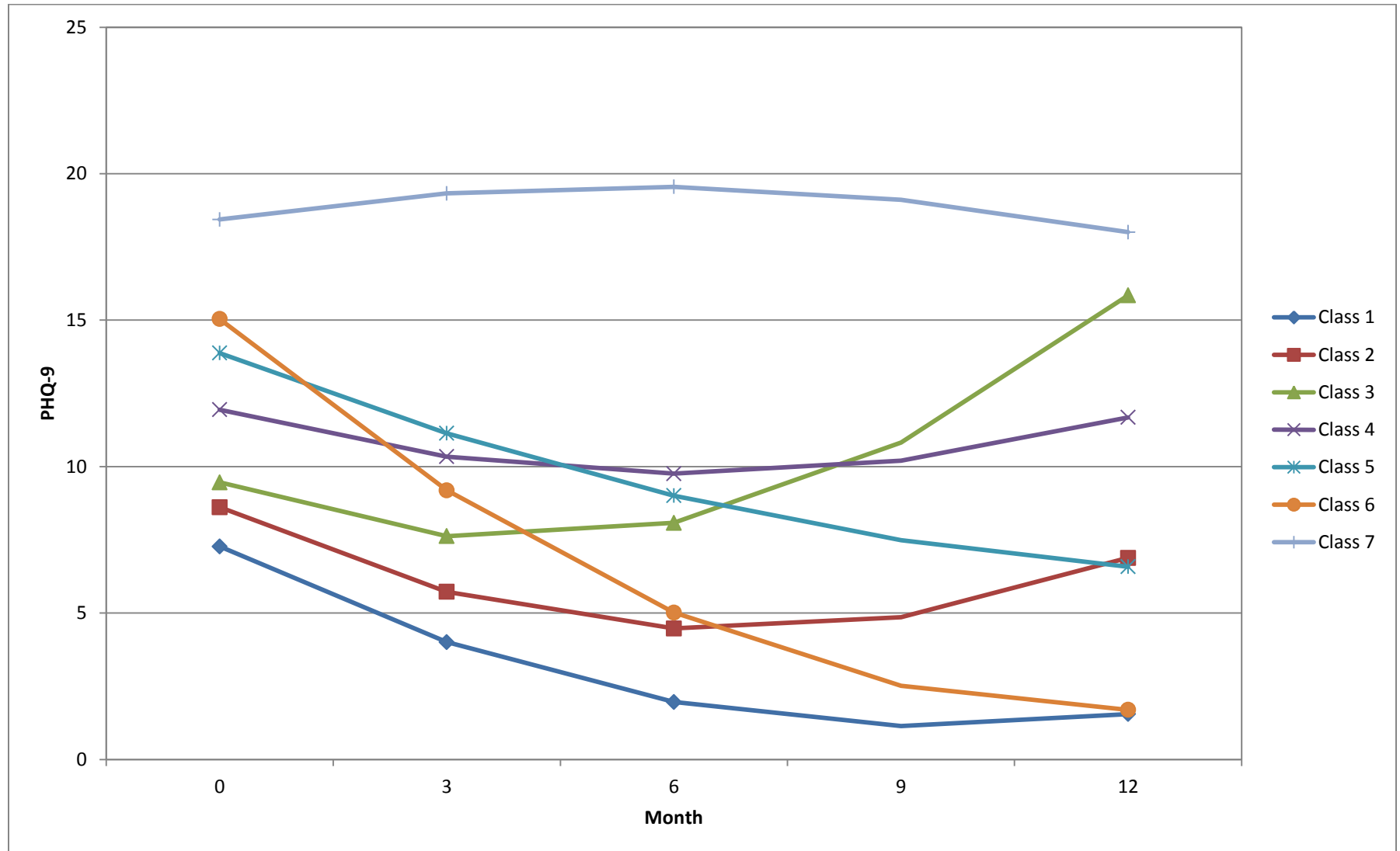

Supplement: S3 Fig — Based on the baseline PHQ-9 scores, one class started with mild depressive symptoms, four classes started with moderate depressive symptoms and two classes started with moderately severe depressive symptoms. (PDF) [file pone.0147775.s003.pdf]
