# Supplementary material for: Trajectory Pathways for Depressive Symptoms and Their Associated Factors in a Chinese Primary Care Cohort by Growth Mixture Modelling
Source: PLoS One. 2016 Feb 1;11(2):e0147775. doi: 10.1371/journal.pone.0147775 (PMC4734622; doi:10.1371/journal.pone.0147775)
Supplement: S2 Fig — The seven-class model has the lowest BIC (13597.25) and Consistent AIC (13629.25), and the second lowest AIC (13450.67) and Sample Adjusted BIC (13495.64). The indicators of Goodness-of-fit statistics tended to level-off after seven-class model, suggesting that the best fitting model was the seven-class model. Hence, seven classes were retained as the most better-fit model. (PDF) [file pone.0147775.s002.pdf]

Figure 2: Figure of the Elbow Plot of Goodness-of-fit statistics

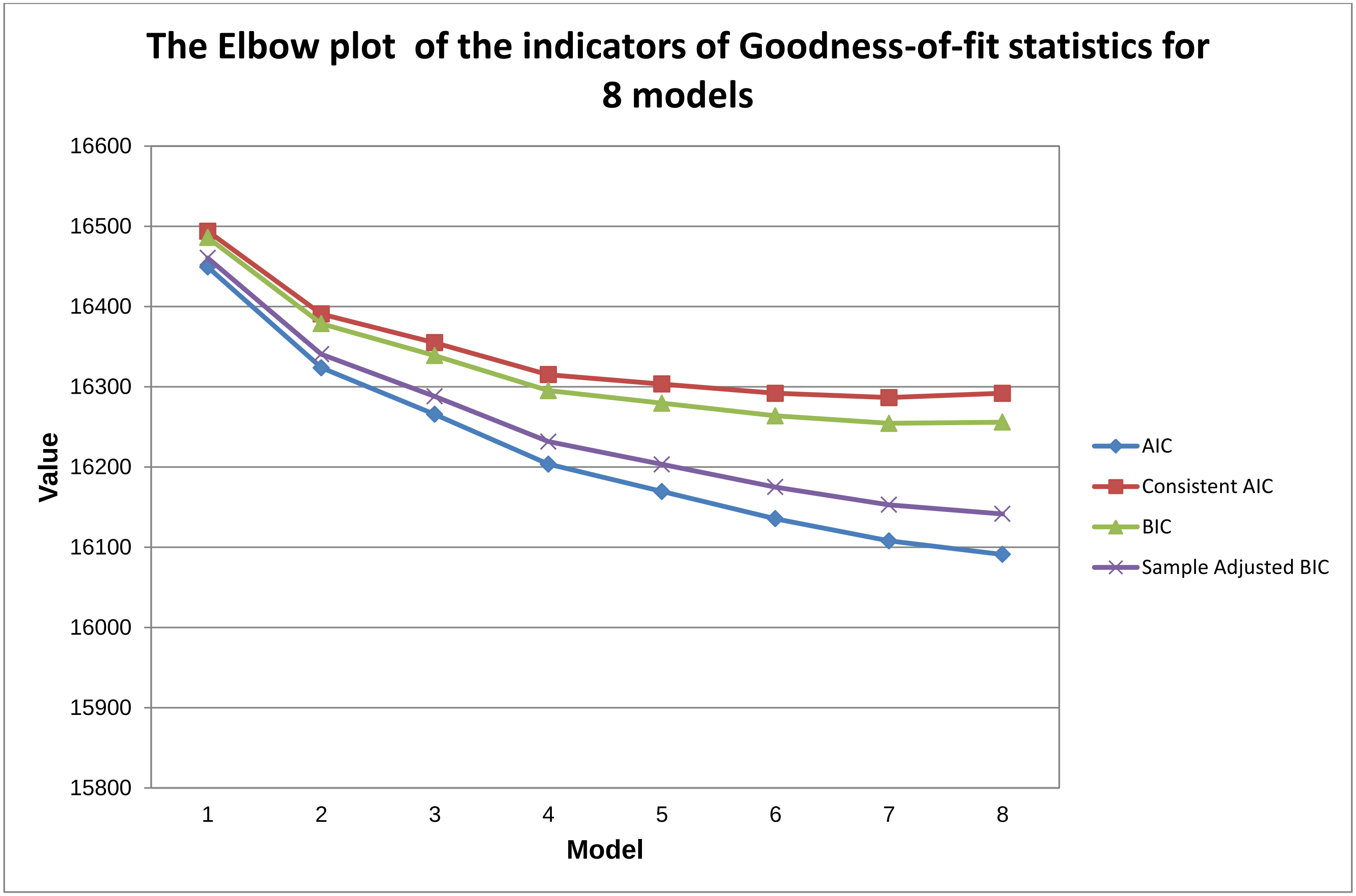

AIC: Akaike Information Criterion

BIC: Bayesian Information Criterion
